# Notch signaling promotes a HIF2α-driven hypoxic response in multiple tumor cell types

**DOI:** 10.1038/s41388-018-0400-3

**Published:** 2018-07-11

**Authors:** Anders P. Mutvei, Sebastian K.-J. Landor, Rhys Fox, Eike-Benjamin Braune, Yat Long Tsoi, Yee Peng Phoon, Cecilia Sahlgren, Johan Hartman, Jonas Bergh, Shaobo Jin, Urban Lendahl

**Affiliations:** 1 Department of Cell and Molecular Biology, Karolinska Institutet, Stockholm, Sweden; 2 Turku Centre for Biotechnology, University of Turku and Åbo Akademi University, 20520 Turku, Finland; 3 Department of Oncology-Pathology, Karolinska Institutet, Stockholm, Sweden; 4 Department of Oncology-Pathology, Karolinska Institutet, Radiumhemmet, Breast Cancer, Cancer Theme, Karolinska University Hospital, Stockholm, Sweden; 5 Present Address: Department of Public Health, Oxford University, Oxford, OX1 2JD UK

## Abstract

Hyperactivation of Notch signaling and the cellular hypoxic response are frequently observed in cancers, with increasing reports of connections to tumor initiation and progression. The two signaling mechanisms are known to intersect, but while it is well established that hypoxia regulates Notch signaling, less is known about whether Notch can regulate the cellular hypoxic response. We now report that Notch signaling specifically controls expression of HIF2α, a key mediator of the cellular hypoxic response. Transcriptional upregulation of HIF2α by Notch under normoxic conditions leads to elevated HIF2α protein levels in primary breast cancer cells as well as in human breast cancer, medulloblastoma, and renal cell carcinoma cell lines. The elevated level of HIF2α protein was in certain tumor cell types accompanied by downregulation of HIF1α protein levels, indicating that high Notch signaling may drive a HIF1α-to-HIF2α switch. At the transcriptome level, the presence of HIF2α was required for approximately 21% of all Notch-induced genes: among the 1062 genes that were upregulated by Notch in medulloblastoma cells during normoxia, upregulation was abrogated in 227 genes when HIF2α expression was knocked down by HIF2α siRNA. In conclusion, our data show that Notch signaling affects the hypoxic response via regulation of HIF2α, which may be important for future cancer therapies.

## Introduction

Interaction between signaling pathways is vital during normal development and tissue homeostasis. Dysregulation of signaling pathways is also increasingly linked to cancer and a downside of pathway integration is that dysregulation of a particular pathway in a tumor situation may also influence signaling from other interacting pathways, further aggravating disease. An improved understanding of how signaling pathways interact is therefore warranted, as it may facilitate tailored therapy approaches based on identified pathway abnormalities.

In this study, we addressed whether the Notch singling pathway modulates the cellular response to hypoxia, i.e., low oxygen conditions. The Notch signaling pathway is a highly evolutionarily conserved cell-cell contact-dependent signaling mechanism, which is activated when a ligand binds to a Notch receptor, leading to receptor cleavage and the release of the Notch intracellular domain (Notch ICD). Notch ICD subsequently translocates to the nucleus and forms a ternary transcriptional activation complex with CSL (also known as RBP-Jk) and Mastermind-like (MAML) to induce expression of downstream target genes, including Notch-regulated ankyrin repeat-containing protein (NRARP), Hes, or Hey genes [1, 2]. Notch mutations are found in several tumor types, having either oncogenic or tumor suppressor roles, depending on the type of tumor [3].

In order to adapt their physiological responses to different oxygen levels, cells are endowed with a specific signaling system: the cellular hypoxic response. Central to the cellular hypoxic response are the two oxygen-labile transcription factors: Hypoxia-inducible factor (HIF) 1α and 2α (collectively referred to as HIFα). In normoxia, HIFα is hydroxylated by oxygen-sensing prolyl hydroxylase proteins, leading to ubiquitylation by the E3 ubiquitin ligase Von Hippel-Lindau (VHL) and subsequent proteasomal degradation. Under hypoxic conditions, the prolyl hydroxylases are inactivated, resulting in stabilization of HIFα, which bind to the constitutively expressed HIF1β and activate downstream target genes [4]. Although HIF1α and HIF2α are structurally quite similar [5], they exert at least partly different functions by activating genes specific to each paralog [6–10] (for review see [11]); for example, HIF1α controls genes involved in glycolysis, whereas HIF2α regulates matrix metalloproteases important for cellular motility and invasion [6, 8,12–14]. HIF1α and HIF2α also exhibit different temporal patterns upon a hypoxic onset in certain contexts. In neuroblastoma, HIF1α is stabilized rapidly in response to hypoxia, mediating the acute cellular response to oxygen deprivation, whereas HIF2α accumulates later and mediates the chronic effects of hypoxia [15, 16]. The transition from HIF1α to HIF2α is referred to as the HIF1α-to-HIF2α switch [17], but the molecular basis for this transition remains poorly understood. Hypoxia signaling components are frequently mutated in cancers. Abnormal HIF2α stabilization, through HIF2α gain-of-function or VHL loss-of-function mutations [17], has been found in pheochromocytomas and paragangliomas [18–20], as well as loss of VHL in clear cell renal carcinoma (for review see [21, 22]). Furthermore, hypoxic tumors promote resistance to chemotherapy and radiation treatment (for review, see [23]).

Upon hypoxia, Notch signaling activity is increased through multiple mechanisms [24]. HIF1α directly binds to and stabilizes Notch ICD [25, 26] during hypoxia, leading to enhanced activation of Notch downstream genes [27–31]. Hypoxia also induces expression of Notch ligands, such as Jagged2 and Delta-like Ligand 4 (Dll4) [32–35]. In contrast, whether Notch signaling influences the cellular hypoxic response remains less explored [36–38]. Here, we report that Notch signaling regulates the hypoxic response in multiple tumor types by controlling HIF2α expression. In addition, we provide evidence that a significant portion of the Notch-induced transcriptome requires functional HIF2α.

## Results

### Notch signaling upregulates HIF2α mRNA levels in various types of cancer cells

**Fig. 1:**
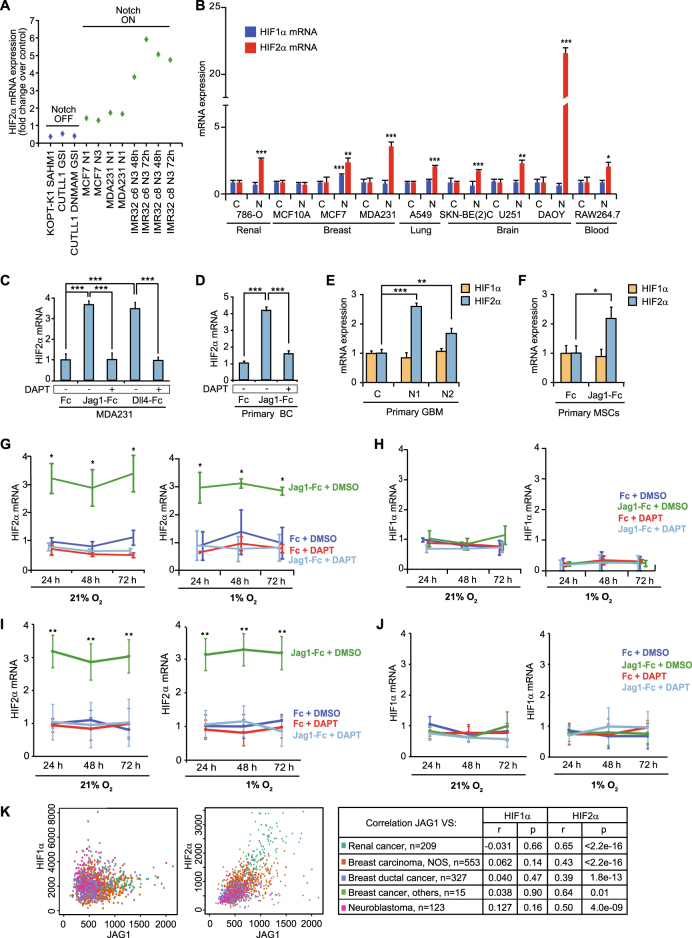
Notch signaling activates HIF2α expression. **a** HIF2α expression levels in publicly available transcriptome data sets where Notch signaling had been blocked (=Notch OFF) by the use of γ-secretase inhibitors (GSI), a dominant negative version of MAML1 (DNMAM) or by a stapled Maml1 peptide (SAHM1), or activated (=Notch ON) by Notch 1 or Notch 3 ICD (N1 and N3, respectively). **b** HIF1α and HIF2α mRNA expression measured by quantitative PCR (qPCR) in nine cell lines infected with adenoviral vectors expressing GFP (C; control) or Notch1 ICD (N) for 24 h at normoxia (see Supplementary Figure 1A for Notch1 ICD expression levels). **c, d** HIF2α mRNA expression levels measured by qPCR in MDA-MB-231 breast cancer cells (**c**) or primary breast cancer cells (**d**), cultured for 24 h at normoxia on immobilized Jagged1 (Jag1-Fc) or Dll4 ligands (Dll4-Fc), or Fc fragments as control, in combination with the γ-secretase inhibitor DAPT, as indicated. **e, f** HIF1α and HIF2α mRNA expression in primary glioblastoma (GBM) cells (**e**) or primary mesenchymal cells (MSCs) (**f**) after 24 h of Notch activation by adenovirus-mediated expression of Notch1 ICD (N1) or Notch2 ICD (N2), (**e**) or by immobilized Jagged1 ligand (**f**). (**G–J**) HIF2α (**G, I**) and HIF1α (**H, J**) mRNA expression measured by qPCR in MDA-MB-231 (**g, h**) or primary breast cancer cells (**i, j**), cultured at normoxia (21% O_2_) or hypoxia (1% O_2_) for 24, 48, and 72 h, on immobilized Jagged1 (Jag1-Fc) ligands, or Fc fragments as control, in combination with DMSO or DAPT, as indicated. (**K**) Correlation between Jagged1 (JAG1) and HIF1α or HIF2α mRNA expression levels in transcriptome data sets from the GeneSapiens data base. *r* = correlation coefficient. *p* = *p* value. Values are significant at *** *p* < 0.001, **p < 0.01, and *p < 0.05. Graphs represent an average of at least three independent experiments

To learn whether Notch signaling affects the cellular hypoxic response, we searched published Notch-transcriptomes for alterations in expression of genes involved in the hypoxic response. In several transcriptomes, we noted that HIF2α mRNA expression levels were increased during conditions where Notch signaling was activated and, conversely, decreased under conditions of Notch blockage (Fig. 1a). To test whether Notch regulates HIF2α mRNA expression, we expressed an activated form of Notch (Notch1 ICD) (Supplementary Figure 1A) in nine different human tumor cell lines derived from a range of tumors (renal, breast, lung, brain, and blood cancer cells), and monitored HIF1α and HIF2α mRNA levels in normoxia (21% oxygen). HIF2α mRNA levels were significantly upregulated by Notch activation in eight of the nine cell lines in normoxia (Fig. 1b). In contrast, expression of HIF1α remained unchanged in all cell lines except for in the estrogen receptor-positive cell line MCF7 (Fig. 1b). As a control for Notch activation, expression levels of the canonical Notch target genes NRARP, Hes1, and Hey1 [39] were shown to be upregulated in all cell lines (Supplementary Figure 1B). To learn whether a more physiological mode of Notch activation can induce HIF2α expression, we cultured MDA-MB-231 cells on immobilized Jagged1 or Dll4 ligands in normoxia. Ligand stimulation in both cases gave rise to a robust activation of HIF2α mRNA expression, which was abrogated by the γ-secretase inhibitor 5-difluorophenylacetyl-L-alanyl-2-phenylglycine-1,1-di-methylethyl ester (DAPT) (Fig. 1c). Thus, HIF2α activation can be induced by Notch signaling in a process requiring the release of the Notch ICD.

We next investigated if Notch signaling controls HIF2α also in cells from primary human cancers. Primary breast cancer and glioblastoma cells robustly upregulated HIF2α mRNA expression upon Notch activation in normoxia, using Jagged1 ligand stimulation or expression of Notch1 and 2 ICD (Fig. 1d, e). In addition, Notch-activated HIF2α mRNA also in non-tumorigenic primary mesenchymal cells (Fig. 1f). Taken together, these data demonstrate that elevated Notch signaling induces HIF2α mRNA expression in several established cell lines and primary tumor cells in normoxia.

To explore whether a similar relationship between Notch and HIF2α was observed also under hypoxia, we monitored mRNA levels of HIF1α, HIF2α, and NRARP in MDA-MB-231 cells cultured on immobilized ligands for 24, 48, or 72 h in normoxia or hypoxia. To avoid potential pericellular hypoxia from high cellular confluence, sparse seeding was used for later time points (Supplementary Figure 1C). HIF2α and NRARP mRNA expression was upregulated already after 24 h of Notch signaling activation, and remained significantly elevated at all time points under both hypoxic and normoxic conditions (Fig. 1g and Supplementary Figure 1D). The elevated levels of HIF2α and NRARP expression by Notch were dependent on Notch receptor cleavage, as the upregulation was abrogated by DAPT treatment (Fig. 1g and Supplementary Figure 1D). In contrast, HIF1α mRNA levels were not altered by changes in Notch signaling, but were decreased by hypoxia at all time points analyzed (Fig. 1h). An equivalent robust upregulation of HIF2α, but not of HIF1α, was observed in primary breast cancer cells (Fig. 1i, j).

Given that Notch controls HIF2α expression in multiple tumor cell types, we investigated whether the level of Notch signaling correlated with HIF2α expression levels across primary cancers, using transcriptome data from multiple tumors of renal, breast, neuroblastoma, and medulloblastoma origin [40]. Since Notch downstream target genes are known to vary in a cell context-dependent manner [1, 41], we utilized Jagged1 expression as a proxy for active Notch signaling, in keeping with previous studies [42, 43]. HIF2α and Jagged1 expression correlated significantly in all tumor types analyzed (renal cancer, *n* = 209, *R*^2^ = 0.65, *p* < 2.2 * 10^−16^; breast carcinoma, not otherwise specified (NOS), *n* = 553, *R*^2^ = 0.43, *p* < 2.2*10^−^^16^; ductal breast cancer, *n* = 327, *R*^2^ = 0.39, *p* < 1.8 * 10^−^^13^, breast cancer, others, *n* = 15, *R*^2^ = 0.64, *p* < 0.01; neuroblastoma, *n* = 123, *R*^2^ = 0.50, *p* < 4.0 * 10^−^^9^; medulloblastoma, *n* = 62, *R*^2^ = 0.36, *p* = 0.0043; Fig. 1k, Supplementary Figure 1E). In contrast, HIF1α and Jagged1 expression levels did not correlate significantly in any of the tumors (renal cancer; *R*^2^ = −0.031, *p* = 0.66; breast carcinoma, NOS, *R*^2^ = 0.062, *p* = 0.14; breast ductal cancer, *R*^2^ = 0.040, *p* = 0.47; breast cancer, others, *R*^2^ = 0.038, *p* = 0.90; neuroblastoma, *R*^2^ = 0.127, *p* = 0.16; medulloblastoma, *R*^2^ = 0.04, *p* = 0.73; Fig. 1k, Supplementary Figure 1E). In sum, these data show that Notch signaling regulates HIF2α mRNA levels in a variety of cancers.

### HIF2α mRNA expression is regulated by canonical Notch signaling but not through direct transcriptional activation via the HIF2α proximal promoter

**Fig. 2:**
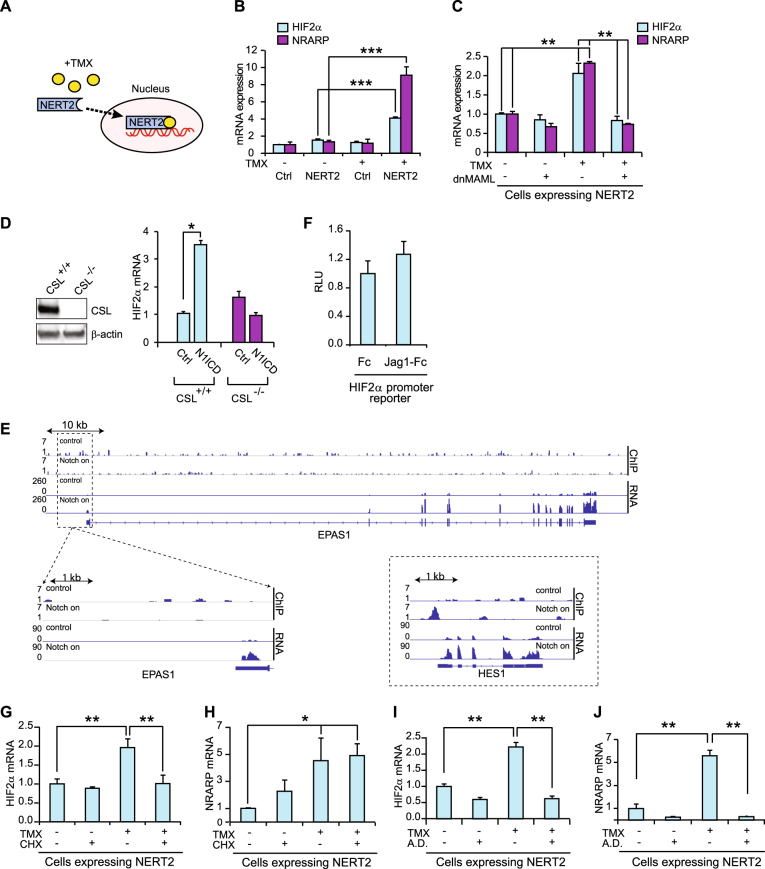
HIF2α expression is regulated by canonical Notch signaling, but not through direct transcriptional activation. **a** Schematic illustration of the NERT2 system where Notch1 ICD nuclear localization and thus Notch signaling is activated by addition of 4-OH tamoxifen (TMX). **b, c** HIF2α and NRARP mRNA expression after Notch signaling activation by 50 nM TMX in DAOY cells transiently (**b**) or stably (**c**) expressing NERT2, in combination with expression of dnMAML **(c)**. **d** Western blot analysis of CSL levels in CRISPR/Cas9-inactivated (CSL^-/-^) and control (CSL^+/+^) DAOY cells (left), and with HIF2α mRNA expression upon Notch1 ICD expression in these cells (right). **e** Analysis of CSL binding to the promoters of HIF2α (EPAS1; top and lower left) and HES1 (lower right) under control or Jagged1-stimulating conditions (Notch on). No specific CSL binding was recorded in the HIF2α promoter, whereas CSL binding to an established CSL-binding site located approximately 60 bp of the HES1 transcription start site was recorded following Notch activation. **f** Analysis of a HIF2α promoter-luciferase reporter construct upon cultivation of MDA-MB-231 cells on immobilized Jagged1 ligand (Jag1-Fc) or Fc fragments. **g–j** Analysis of HIF2α **(g, i)** and NRARP **(h, j)** mRNA expression in DAOY cells transiently expressing NERT2 in combination with TMX treatment to activate Notch signaling. Cells were treated with 10 µg/mL cycloheximide (CHX) for 8 h to block translation **(g, h)** or 1 µg/mL actinomycin D (A.D.) for 8 h to block transcription **(i, j)**. Values are significant at *** *p* < 0.001, **p < 0.01, and *p < 0.05. Graphs represent an average of at least three independent experiments

Canonical Notch signaling is executed via the ternary Notch ICD/MAML1/CSL complex acting on the promoter of a downstream gene, yet various forms of non-canonical signaling exist, some of which bypass the need for nuclear localization of Notch [44]. To learn whether nuclear localization of Notch1 ICD is required for HIF2α activation, we transiently expressed a Notch1 ICD-estrogen receptor fusion construct (NERT2), which relocates from the cytoplasm to the nucleus upon addition of tamoxifen [42, 45, 46] (Fig. 2a). In medulloblastoma DAOY cells, as well as in MCF7 cells, expression of cytoplasmic NERT2 did not enhance HIF2α or NRARP expression, whereas tamoxifen-induced nuclear localization of NERT2 for 8 h led to a robust upregulation of HIF2α and NRARP expression (Fig. 2b, Supplementary Figure 2A). To test whether the canonical Notch transcriptional activation complex is required for HIF2α upregulation, we introduced a truncated form of MAML1 (dnMAML) that blocks the interaction between Notch ICD and CSL [47]. Expression of dnMAML abrogated NERT2-mediated upregulation of both HIF2α and NRARP expression following tamoxifen treatment (Fig. 2c, Supplementary Figure 2B), indicating that HIF2α expression is controlled via canonical Notch signaling. In support of this notion, inactivation of the CSL gene by CRISPR/Cas9 genome editing also blunted the upregulation of HIF2α by Notch in DAOY cells (Fig. 2d).

To learn more about how Notch upregulates HIF2α, we investigated whether the canonical Notch transcriptional machinery binds directly to the HIF2α promoter, or whether upregulation of HIF2α occurs in a more indirect fashion. To test the first scenario, we conducted a chromatin immunoprecipitation sequencing (ChIP-seq) experiment to analyze potential binding of CSL to the HIF2α (EPAS1) locus. No specific CSL occupancy was detected in the HIF2α promoter or gene during Notch activation under which CSL binding to a well-established binding site in the HES1 promoter was detected (Fig. 2e). In keeping with this finding, Notch activation did not stimulate a HIF2α promoter-luciferase reporter construct containing the −1000 to +418 bp region flanking the transcriptional start site (Fig. 2f). To explore if HIF2α expression was regulated in a more indirect manner, we tested the effect of blocking protein translation by cycloheximide (CHX) in conjunction with Notch activation. CHX treatment abrogated the Notch-dependent upregulation of HIF2α, whereas the upregulation of NRARP was unaffected by CHX (Fig. 2g, h), as expected. Blockage of transcription by actinomycin D treatment abrogated Notch activation of both HIF2α and NRARP (Fig. 2i, j). Collectively, these data suggest that Notch ICD/CSL activates an intermediate protein controlling the HIF2α upregulation, rather than binding to the HIF2α promoter. Of note, actinomycin D negatively regulated mRNA expression of NERT2 (Supplementary Figure 2C, D), but the strong induction of NRARP expression (Fig. 2b, c, h) argues that sufficient amounts of the NERT2 protein are “preloaded” in the cytoplasm prior to tamoxifen induction to execute a robust Notch activation. Induction by tamoxifen increased the expression of NERT2 mRNA (Supplementary Figure 2C, D), but the reason for this is not understood. We next tested the potential role of three candidate intermediate proteins in the Notch-activation of HIF2α (Hes1, Hey1, and p53), which in other contexts have been shown to mediate Notch responses [42]. Overexpression of wild-type p53 (since p53 is mutated in MDA-MB-231 cells), Hes1 or Hey1, however, did not upregulate HIF2α (Supplementary Figure 2E). Collectively, these data suggest that HIF2α is controlled by canonical Notch signaling in an indirect manner.

### Elevated Notch signaling enhances HIF2α protein levels and induces a HIF1α-to-HIF2α switch

**Fig. 3:**
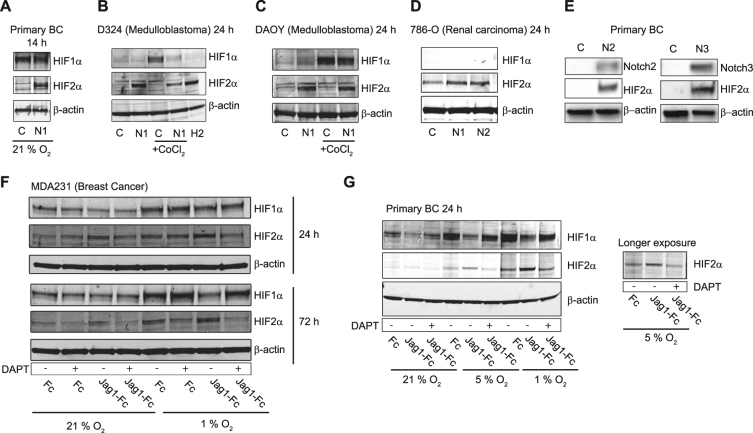
Notch signaling increases HIF2α protein expression and triggers a HIF1α-to-HIF2α switch. **a–g** HIF1α and HIF2α protein levels measured by Western blotting in: (**a, e, g**) primary breast cancer cells, (**b**) D324 medulloblastoma cells, (**c**) DAOY medulloblastoma cells, (**d**) 786-O renal carcinoma cells and (**f**) MDA-MB-231 cells, cultured on immobilized Jagged1 ligands in combination with DAPT treatment (**f, g**) or upon adenovirus-mediated expression of GFP (**c**), Notch1 ICD (N1), Notch2 ICD (N2), or Notch3 ICD (N3) (**b, c, d, e**), as indicated. In **(b)** and **(c)**, cells were treated with *cobalt* (II) *chloride* (CoCl_2_) as a hypoxia mimetic and in **(f)** and **(g)**, cells were cultured in 21, 5, or 1% oxygen, as indicated. **b** H2 = transient expression of HIF2α from an exogenous promoter as positive control. All experiments were repeated at least three times

The HIFα proteins are degraded in normoxia, but given the magnitude of the HIF2α mRNA induction by Notch, we reasoned that the increase in mRNA may result in increased HIF2α protein levels also in normoxia. In line with this, ectopic Notch1 ICD expression, which results in high levels of Notch signaling activation, increased normoxic HIF2α protein in primary breast cancer cells (Fig. 3a), human medulloblastoma cell lines D324 and DAOY (Fig. 3b, c), as well as the VHL-deficient 786-O renal carcinoma cell line (Fig. 3d). The ability of Notch to activate HIF2α is conserved across the different Notch paralogs, as Notch2 and 3 ICDs also induced robust HIF2α protein upregulation (Fig. 3e). Ligand-mediated activation, which produces a more moderate Notch signaling activation, readily increased HIF2α protein in normoxia in MDA-MB-231 after 24 h, but not 72 h, of activation (Fig. 3f), and did not elevate normoxic HIF2α protein in primary breast cancer cells (Fig. 3g). However, if breast cancer cells were ligand-stimulated during hypoxia, increased HIF2α protein levels were observed, both in primary breast cancer cells and MDA-MB-231 cells (Fig. 3f, g). Elevated HIF2α protein levels were also observed when D324 and DAOY cells were treated with CoCl_2_, a widely used hypoxia mimetic (Fig. 3b, c).

In several of the cell types, Notch activation also led to decreased HIF1α protein levels: this was observed in D324 cells (Fig. 3b) and primary breast cancer both at normoxia and hypoxia (Fig. 3g), as well as in the MDA-MB-231 cells after 72 h in hypoxia (Fig. 3f). The fact that HIF1α downregulation was observed in the primary breast cancer cells after 24 h (Fig. 3g), but not after 14 h (Fig. 3a) may indicate that it takes a certain time period to achieve this downregulation and execute the HIF1α-to-HIF2α switch. A number of proteins have been implicated in controlling the HIF1α-to-HIF2α switch, including HAF [17], HSP70, and CHIP [48]. While HAF and HSP70 were not regulated by Notch1 ICD (data not shown), we found that CHIP, which encodes a ubiquitin ligase involved in HIF1a degradation [48], was moderately upregulated by Jagged1 immobilized ligand stimulation and strongly reduced by DAPT (Supplementary Figure 3A), making it a candidate mediator between Notch and HIF1α regulation.

### A subset of the Notch-induced transcriptome depends on the presence of HIF2α

**Fig. 4:**
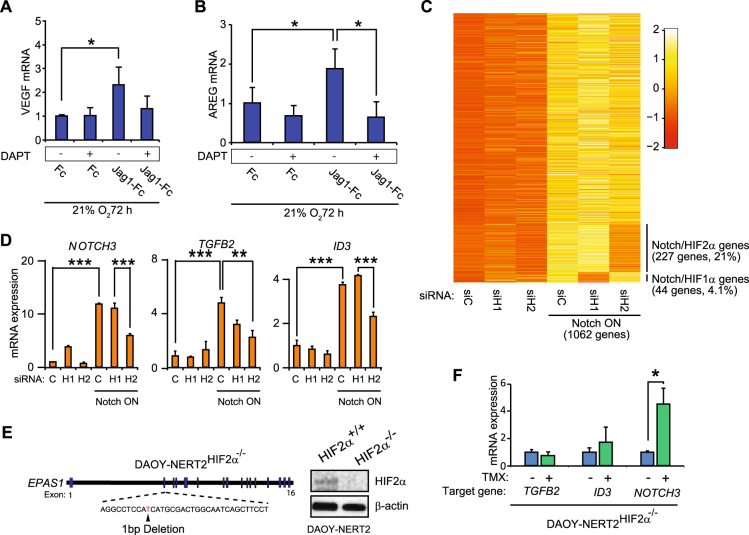
HIF2α is required for a portion of the Notch-induced transcriptome to be activated. (**a, b)** VEGF **(a)** and AREG **(b)** mRNA expression in MDA-MB-231 breast cancer cells cultured on immobilized Jagged1 ligands (Jag1-Fc), or control fragments (Fc), cultured at 21% O_2_ for 72 h in combination with DAPT treatment, as indicated. **c** Heatmap of Notch-activated genes in DAOY-NERT2 cells subjected to either HIF1α (H1) or HIF2α (H2) siRNA-knockdown (see also Supplementary Figure 4). A total of 1062 genes were upregulated by activated Notch signaling (Notch ON) in normoxia, of which 227 genes were downregulated by HIF2α siRNA knockdown, and 44 genes by HIF1α siRNA knockdown. **d** mRNA expression levels, measured by qPCR, of three genes (*NOTCH3*, *TGFB2*, and *ID3*) from **(c**). **e** Schematic representation of the CRISPR/Cas9 targeting strategy for the HIF2α gene (*EPAS1*) to generate DAOY-NERT2^HIF2α−/−^ cells, with a Western blot showing targeting efficiency. **f** mRNA expression levels, measured by qPCR, of *NOTCH3*, *TGFB2*, and *ID3* in DAOY-NERT2^HIF2α−/−^ cells with and without tamoxifen treatment (TMX) to activate Notch signaling. Values are significant at *** *p* < 0.001, **p < 0.01, and *p < 0.05. Graphs represent an average of at least three independent experiments

Given that HIF2α is a transcription factor, a potential consequence of our findings is that a Notch-induced transcriptome may consist of genes that are activated by both Notch ICD and HIF2α. To learn whether HIF2α-specific genes are induced during Notch activation, we first tested whether Notch increased expression of two genes, VEGF and AREG, which are regulated by hypoxia and HIF2α, respectively [49, 50]. Both VEGF and AREG were upregulated by Notch in MDA-MB-231 cells cultured in normoxia, and activation was abrogated by DAPT (Fig. 4a, b).

To assess how Notch induction of HIF2α affected gene expression globally, we activated Notch1 signaling by tamoxifen in DAOY cells stably expressing NERT2 (DAOY-NERT2) for 48 h under normoxia and hypoxia, in combination with siRNA-mediated depletion of HIF1α or HIF2α (Supplementary Figure 4A, B). Genome-wide RNA-sequencing analysis revealed that 1062 genes were upregulated upon Notch activation in normoxia, 881 genes in hypoxia, and 547 genes were upregulated by Notch in both normoxia and hypoxia, including the canonical Notch targets NRARP, HES4, and HEY1 (Supplementary Figure 4C). A total of 227 of the 1062 genes (21%) induced by Notch in normoxia were downregulated by HIF2α siRNA knockdown (Fig. 4c, Supplementary File 1). In contrast, only 44 of the 1062 Notch-activated genes (4.1%) were downregulated by HIF1α knockdown (Fig. 4c, Supplementary File 1). These data suggest that a substantial proportion of the normoxic Notch transcriptome in medulloblastoma requires HIF2α, but not HIF1α, for activation. A small set of genes (4.7%) was also regulated both by HIF1α and HIF2α (Supplementary File1). Enrichment analysis of functional annotation Gene Ontology terms revealed that several of the 227 Notch-activated and HIF2α-dependent genes are involved in cell adhesion, blood vessel development, and signal transduction (Supplementary table 1). qPCR validation of three of these genes, Notch3, TGFB2, and ID3, showed a normoxic Notch induction, which was blunted by siRNA to HIF2α (Fig. 4d). Similarly, in DAOY-NERT2 HIF2α knockout cells (Fig. 4e), TGFB2 and ID3 expression was not upregulated by tamoxifen-induced activation of Notch1 ICD, and upregulation of Notch3 was reduced (Fig. 4f) as compared to a level comparable to that obtained by siRNA for HIF2α in Fig. 4d. Overall, these data suggest that a substantial subset of the Notch-induced transcriptome requires functional HIF2α.

### Notch receptor paralog-specific effects on medulloblastoma tumor growth

**Fig. 5:**
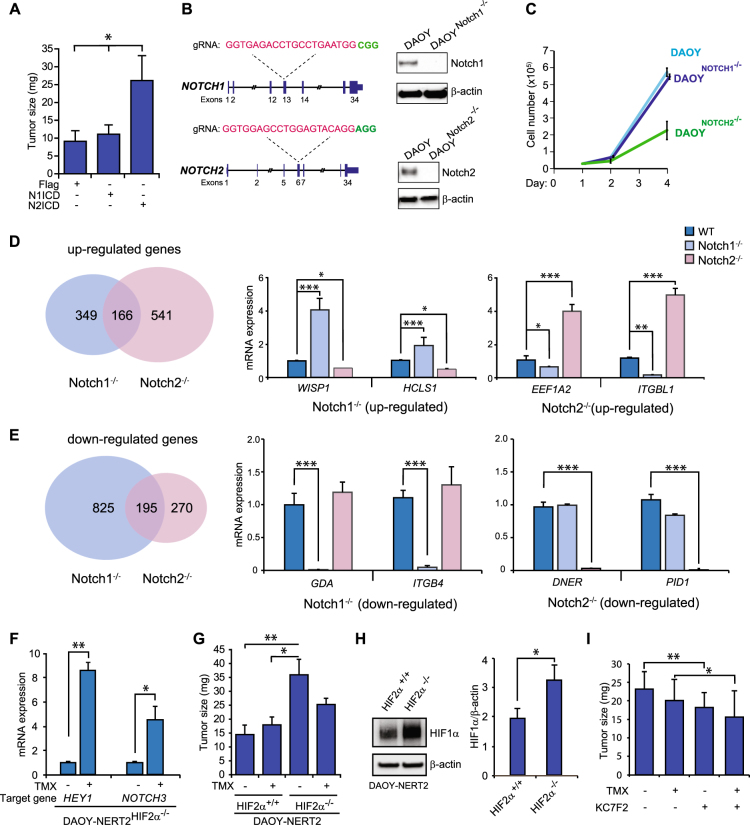
Notch1 and 2 ICD exert distinct effects on tumor growth. **a** Analysis of tumor growth in the chick chorioallantoic membrane (CAM) assay following overexpression of Notch1 or 2 ICD, as indicated. **b** Schematic representation of the CRISPR/Cas9 targeting strategy for the Notch1 and Notch2 genes (left) and protein levels measured by Western blotting in DAOY, DAOY^Notch1−/^^−^, and DAOY^Notch2^^−^^/^^−^ cells. **c** Analysis of cell growth for DAOY, DAOY^Notch1−/^^−^, and DAOY^Notch2^^−^^/^^−^ cells. **d, e** VENN diagrams describing differentially expressed genes between DAOY^Notch1−/^^−^ and DAOY^Notch2^^−^^/^^−^ cells, as compared to DAOY control cells, from transcriptomic analysis of these cells (left). Examples of genes specifically upregulated in DAOY^Notch1−/^^−^ (*WISP1* and *HCLS1*) and DAOY^Notch2^^−/−^ (*EEF1A2* and *ITBGL1*) cells (**d**), or downregulated in DAOY^Notch1−/^^−^ (*GDA* and *ITGB4*)) and DAOY^Notch2−^^/^^−^ (*DNER* and *PID1*) cells (**e**) are shown to the right. **f** mRNA expression levels measured by qPCR for *HEY1* and *NOTCH3* in DAOY-NERT2^HIF2α−^^/^^−^ cells treated with a tamoxifen to activate Notch signaling. **g** Analysis of tumor growth of Notch1-activated DAOY-NERT2^HIF2α+/+^ or DAOY-NERT2^HIF2α−^^/^^−^ cells, using the CAM assay. **h** Western blot of HIF1α protein levels in DAOY ^HIF2α+/+^ and DAOY^HIF2α−^^/^^−^ cells (quantification to the right). **i** Effects on tumor growth of the DAOY-NERT2^HIF2α−^^/^^−^ cells after treatment with the HIF1α inhibitor KC7F2 at 40 μM. Values are significant at *** *p* < 0.001, **p < 0.01, and *p < 0.05. Graphs represent an average of at least three independent experiments

We next assessed how altered Notch and HIF2α signaling impacts on the tumor potential of DAOY cells. We first explored how Notch1 or Notch2 expression affected growth of DAOY cells using the chorioallantoic membrane (CAM) xenograft model [37]. Enhanced tumor growth was observed in the CAM assay after Notch2 ICD, but not Notch1 ICD, expression (Fig. 5a). To further assess Notch paralog specificity, we inactivated Notch1 or Notch2 using CRISPR/Cas9, generating DAOY^Notch1−/−^ and DAOY^Notch2−/−^ cells, respectively (Fig. 5b). Growth rates of DAOY^Notch1−/−^ cells were very similar to that of control DAOY cells, whereas DAOY^Notch2−/−^ cells showed markedly reduced growth (Fig. 5c), corroborating a specific role for Notch2 in medulloblastoma tumor growth [51]. Transcriptomic analysis of the DAOY^Notch1−/−^ and DAOY^Notch2/-^ cells revealed 166 genes that were upregulated in both DAOY^Notch1−/−^ and DAOY^Notch2−/−^ cells, but also 541 genes specifically upregulated in the DAOY^Notch2−/−^ cells (including *EEF1A2* and *ITGBL1*), and 349 genes specifically upregulated only in the DAOY^Notch1−/−^ cells (including *WISP1* and *HCLS1*) (Fig. 5d; Supplementary File 2). A total of 195 genes were downregulated in both DAOY^Notch1−/−^ and DAOY^Notch2−/−^ cells, while 270 genes were specifically downregulated in the DAOY^Notch2−/−^ cells (including *DNER* and *PID1*), and 825 genes (including *GDA* and *ITGB4*) were downregulated only in the DAOY^Notch1−/−^ cells (Fig. 5e, Supplementary File 2). In sum, these data corroborate the notion that Notch1 and Notch2 play different roles in medulloblastoma tumor growth.

To study how changes in HIF2α signaling affect growth of DAOY cells, we induced Notch1 signaling in DAOY-NERT2 cells ablated for HIF2α (DAOY-NERT2^HIF2α−/−^ cells; see Fig. 4e for HIF2α targeting) or in DAOY-NERT^HIF2α+/+^ control cells. Tamoxifen induction of Notch1 ICD in DAOY-NERT2^HIF2α+/+^ cells resulted in upregulation of *HEY1* and *NOTCH3* (Fig. 5f), and did not promote tumor growth (Fig. 5g), in keeping with the data in Fig. 5a. In contrast, DAOY-NERT2^HIF2α−/−^ cells exhibited more rapid tumor growth than the control cells, which was partially reduced by Notch1 ICD expression by tamoxifen (Fig. 5g). The reason why elevated Notch1 ICD levels specifically led to a decrease in tumor growth from DAOY-NERT2^HIF2α−/−^ cells but not from DAOY-NERT2^HIF2α+/+^ control cells (Fig. 5g) is not understood. It is not likely explained by Notch1 ICD reducing the level of HIF1α, as this was not observed in DAOY cells under hypoxia-mimicking conditions (Fig. 3c). Instead, the large number of genes showing altered expression in response to Notch1-deficiency but not Notch2-deficiency (Fig. 5d, e) may suggest that there exists Notch1-specific growth-suppressing genes, whose functions are unleashed only in the absence of HIF2α.

We next considered the possibility that loss of HIF2α may increase HIF1α protein levels, leading to the promotion of tumor growth. In support of this idea, DAOY-NERT2^HIF2α−/−^ cells exhibited higher HIF1α protein levels under hypoxic conditions (Fig. 5h) and treatment of the DAOY-NERT2^HIF2α−/−^ cells with the HIF1α inhibitor KC7F2 reduced tumor growth both under control and Notch1 ICD-activated conditions (Fig. 5i). In conclusion, these data suggest that the different HIFα and Notch receptor paralogs exert specific effects on the tumor growth of medulloblastoma cells.

## Discussion

Dysregulation of Notch and hypoxia signaling contributes to tumor initiation and development, and it is therefore important to gain molecular insights into how these two signaling mechanisms intersect in a tumor context. It has previously been established that hypoxia regulates Notch signaling activity, but less is known about how changes in Notch signaling activity affect the cellular hypoxic response [24]. In this report, we provide evidence that elevated Notch signaling promotes a HIF2α-driven hypoxic response.

Our data suggest that Notch upregulates HIF2α at the transcriptional level, and that engagement of the Notch1 ICD/MAML1/CSL transcriptional complex is required. This notion is based on the observation that a Notch1 ICD-ER fusion protein (NERT2) did not upregulate HIF2α mRNA levels when Notch1 ICD localized in the cytoplasm, but only after nuclear localization following tamoxifen treatment. Moreover, disruption of the canonical Notch transcriptional activation complex, by inactivation of the CSL gene or expression of dominant negative MAML1, abrogated HIF2α mRNA upregulation. However, Notch1 ICD/MAML1/CSL does not seem to directly activate HIF2α at the proximal promoter level since: (i) the HIF2α mRNA upregulation was sensitive to the protein synthesis inhibitor CHX; (ii) ChIP-seq analysis revealed that there are no DNA sites showing specific CSL occupancy in the HIF2α promoter or gene; and (iii) the HIF2α promoter encompassing 1.5 kb sequence around the HIF2α transcription start site was unresponsive to Notch activation. This indicates the need for a yet unidentified intermediate protein in the Notch-induced HIF2α activation, which would need to be synthesized prior to HIF2α upregulation.

Despite that HIF2α protein normally is degraded in normoxia, HIF2α mRNA upregulation in normoxia led to accumulation of HIF2α protein in several of the tumor cells tested. The degree of HIF2α accumulation is likely linked to the magnitude of the Notch signaling induction, as a strong induction of Notch signaling (by Notch1 ICD expression) led to higher HIF2α protein levels also observed in normoxia, whereas more moderate Notch activation levels, mediated by ligand stimulation, were insufficient to increase HIF2α protein across all cell lines tested during normoxia, although sufficient during hypoxia. This suggests that sufficiently induced levels of HIF2α mRNA and protein in normoxia may override the ubiquitination-mediated degradation machinery. In keeping with this notion, overexpression of HIF2α from an exogenous promoter also resulted in robust HIF2α protein levels at normoxia.

In several of the cell types tested, HIF2α activation by Notch correlated with decreased HIF1α protein levels, suggesting that high levels of Notch signaling induces a HIF1α-to-HIF2α switch. The reciprocal regulation of HIF1α and HIF2α was observed in primary breast cancer cells and in MDA-MB-231 cells after 72 h of Jagged1-stimulation, as well as in Notch-activated medulloblastoma D324 cells when cells were treated with CoCl_2_, a frequently utilized hypoxia-mimetic. A HIF1α-to-HIF2α switch has been previously observed, notably in neuroblastoma cells exposed to prolonged hypoxia [15, 16], but the mechanism behind the switch remains elusive. One possible explanation for the switch is that the Notch-induced rise in HIF2α protein levels triggers the reduction of HIF1α, which would be compatible with our findings described above. However, it should be noted that HIF1α reduction upon induction of HIF2α by Notch was not observed in all cell types tested, for example in DAOY cells (Fig. 3c), although conversely HIF2α depletion by CRISPR/Cas9 resulted in increased amounts of HIF1α protein. Alternatively, reduction of HIF1α levels may elicit an increase in HIF2α levels, or the regulation of the levels of HIF1α and HIF2α may be independently regulated. As Notch regulates HIF2α expression, the first hypothesis, i.e., HIF2α controls the level of HIF1α, may appear likely. Our data may also suggest that Notch activation triggers HIF1α degradation via γ-secretase-dependent upregulation of the ubiquitin ligase CHIP, a mediator of HIF1α degradation. More work is however needed to understand the effects of Notch on the HIF1α-to-HIF2α switch, and why it occurs only in certain cell types.

Through genome-wide transcriptomic analysis of DAOY cells, we found that a substantial portion of the Notch-induced transcriptome in normoxia requires the presence of HIF2α, as approximately 21% of all Notch-induced genes was abrogated by siRNA-mediated knockdown of HIF2α. This contrasted with siRNA-mediated knockdown of HIF1α, which affected the Notch transcriptome to a considerably lesser extent (4.1% of the Notch-induced genes). The most parsimonious explanation for this observation is that Notch upregulates HIF2α, which in turn activates its specific subset of target genes, even under normoxic conditions. This scenario is reminiscent of the relationship between Notch and the transcription factor cMyc, where Notch activation of cMyc leads to complex and overlapping transcriptional responses [52, 53]. A HIF2α-dependent subset of the Notch-induced transcriptome is potentially interesting from a cancer therapy development perspective. There are not yet any functional Notch inhibitors in clinical use [54], but if key tumor-promoting features of hyperactivated Notch signaling would stem from a HIF2α-dependent portion of the Notch transcriptome, blocking HIF2α would represent an interesting alternative approach to curtail the adverse effects of dysregulated Notch signaling. Recently, specific HIF2α inhibitors that bind to a unique cavity in HIF2α were developed [55, 56]. These inhibitors have proven effective in preclinical ccRCC models and in a ccRCC patient [57, 58] (for review see [19]); and it may be interesting to explore their efficacy in blocking aspects of Notch-induced transcriptomes. Additionally, targeting HAF, which is required for the transcriptional activity of HIF2α, may represent an alternative strategy [59]. In conclusion, our data show that Notch signaling regulates HIF2α, a key component in the cellular hypoxic response, and provide an important new facet to how Notch signaling and the cellular hypoxic response interact in tumor cells.

## Materials and methods

### Cell culture

The 786-O, RAW264.7, MCF-7, MDA-MB-231, A549, SKN-BE(2)C, U251, MCF10A, and DAOY (HTB186) cells were purchased from American Type Culture Collection (ATCC). The primary glioblastoma cells were kindly provided by Drs. Monica Nistér and Johan Holmberg (Karolinska Institutet). Anonymized human breast cancer cells were kindly provided by Dr. Johan Hartman (Ethical permit: Regionala Etikprövningsnämnden i Stockholm 2016/937-32). Culture conditions are specified in the Supplementary information.

### Real-time quantitative-PCR analysis

RNA extraction and cDNA synthesis were accomplished as previously described [60]. Real-time PCR analysis was carried out on a 7500 Fast Real-Time PCR system with Fast SYBR Green Master Mix (Applied Biosystems) according to the manufacturer’s recommendations. The primers were synthesized by Eurofin MWG Operon, and sequences are shown in the Supplementary Figure 5. cDNA from Notch-activated primary mesenchymal cells was a kind gift from Dr. Katarina Le Blanc (Karolinska Institutet).

### Western blot analysis and antibodies

For Western blot analysis, cells were lysed in RIPA buffer supplemented with protease inhibitor cocktail (Complete, Roche). Hypoxia treated cells were lysed within the hypoxia chamber. Protein concentration was determined with the Pierce BCA protein assay kit (Thermo Scientific). Protein samples were boiled at 95 °C for 5 min, where after 50 μg was loaded per well in Nu-PAGE 4–12% Bis-Tris gels (Life Technologies) for SDS-PAGE, and then transferred to Protein nitrocellulose membranes (Schleicher and Schuell). Membranes were blocked for 1 h using Odyssey blocking buffer (LI-COR Biosciences), and incubated with the primary antibody, sometimes in combination with the SignalBoost Immunoreaction Enhancer Kit (EMD Millipore), overnight at +4 °C with end-over-end rotation. Immunoblots were visualized with the Odyssey Infrared Imaging system (LI-COR Biosciences) in accordance with the manufacturer’s recommendations. Antibodies are listed in the Supplementary information.

### Activation of Notch signaling by immobilized ligands

Notch activation by immobilized ligands was performed as previously described [61, 62].

### Statistical analysis

Two-sided Student’s *t* test was used to determine if treatments were significantly different from each other, where *p* ≤ 0.05 was considered statistically significant. Error bars represent standard deviation of the mean.

### ChIP-seq analysis

The procedures for CSL chromatin precipitation (ChIP) and the ChIP-seq experiments are provided in the Supplementary information.

## Electronic supplementary material

Supplementary information

Supplementary Figure 1

Supplementary Figure 2

Supplementary Figure 3

Supplementary Figure 4

Supplementary Figure 5

Supplementary Table 1

Supplementary File 1

Supplementary File 2
