# Supplementary material for: Notch signaling promotes a HIF2α-driven hypoxic response in multiple tumor cell types
Source: Oncogene. 2018 Jul 11;37(46):6083–95. doi: 10.1038/s41388-018-0400-3 (PMC6237764; doi:10.1038/s41388-018-0400-3)
Supplement: Supplementary file 1 — Supplementary information [file 41388_2018_400_MOESM1_ESM.pdf]

Supplementary information

Supplementary Figure 1. (A) Protein expression levels for Notch1 ICD (corresponding to Figure 1B). **(B)** NRARP (upper graph), Hes1 and Hey1 (lower graph) mRNA expression levels in cells from Figure 1B, infected with adenoviral vectors expressing GFP (C; control) or Notch1 ICD (N) for 24 hours at normoxia. **(C)** Representative image of cellular confluence from experiments in Figure 1G-H and Figure 3F. **(D)** NRARP mRNA expression levels in MDA-MB-231 breast cancer cells (corresponding to Figure 1G-H), cultured at normoxia (21% O_2) or hypoxia (1% O_2) for 24, 48 and 72 hours, on immobilized Jagged1 (Jag1-Fc) ligands, or Fc fragments as control, in combination with DMSO or DAPT, as indicated. (**E**) Correlation between Jagged1 and HIF1α or HIF2α mRNA expression levels in a medulloblastoma transcriptome data set from [63]. r=correlation coefficient (ρ). p=p value. Values are significant at *** P<0.001, **P<0.01 and *P<0.05. Graphs represent an average of at least three independent experiments.

Supplementary Figure 2. (A-B) HIF2α and NRARP mRNA expression in MCF7 cells transiently expressing NERT2, in combination with 50 nM 4-OH tamoxifen (TMX) to activate Notch signaling **(A)** in combination with expression of dnMAML **(B)**. **(C-D)** mRNA expression of the NERT2 construct in response to actinomycin D (A.D) **(C)** or cycloheximide (CHX) (**D**). **(E)** Expression of HIF2α mRNA in MDA-MB-231 cells transiently expressing wildtype p53, Hes1 or Hey1, or control plasmids (Ctrl).

Supplementary Figure 3. (A) CHIP mRNA expression levels in primary breast cancer cells after culturing on immobilized Jagged1 (Jag1-Fc) ligands, or Fc fragments as control, in combination with DMSO or DAPT, as indicated. **(B)** Representative experiment of the two-color multiplex detection of HIF1α and HIF2α protein that was utilized in Western blot analyses, as described in the Materials and Methods (from Figure 3G).

Supplementary Figure 4. (A-B) HIF1α and HIF2α mRNA levels **(A)** or protein levels **(B)** in DAOY-NERT2 cells cultured with TMX (Notch ON) or without TMX for 48 hours, in combination with siRNA mediated knockdown of HIF1α (H1), HIF2α (H2) or control siRNA (C)

at 21% or 1% O_2, as specified. **(C)** Expression of Notch target genes in the transcriptome analysis of DAOY-NERT2 cells. Values are represented as RPKM, fold-change. Values are significant at *** P<0.001 and **P<0.01. Graphs represent an average of at least three independent experiments.

Supplementary Figure 5. Sequence information for forward (For) and reverse (Rev) primers used in the qPCR experiments.

Supplementary Table 1. GO terms enriched for Notch-HIF2α genes, analyzed by DAVID. Only GO terms with more than 8 genes are shown.

Supplementary File 1. Excel file containing the list of genes that were upregulated or downregulated in the RNA-sequencing analysis of DAOY-NERT2 cells shown in Figure 4C.

Supplementary File 2. Excel file containing the list of genes that were upregulated or downregulated in the RNA-sequencing analysis of the $DAOY^{Notch1-/-}$ and $DAOY^{Notch2-/-}$ cells shown in Figure 5D,E.

Supplementary materials and methods

Culture conditions for cell lines

786-O, RAW264.7, MCF-7, MDA-MB-231, A549, SKN-BE(2)C, U251,DAOY (HTB186), DAOY-NERT2, $DAOY^{Notch1-/-}$ and $DAOY^{Notch2-/-}$ cells were maintained in DMEM with 10 % fetal calf serum and 1% penicillin-Streptomycin (Life Technologies). MCF10A cells were cultured as previously described[1]. The primary glioblastoma cells were maintained in Neurocult Ns-A Basal medium with proliferation supplements: 25 μg/μl human bFGF, 1 μg/μl human EGF (Stem Cell Technologies) and 1 % penicillin-streptomycin (Life Technologies). Anonymized human breast cancer cells were grown in DMEM/F12 supplemented with 5% FBS, 100 ng/ml hFGF, 200 ng/ml hEGF, 5 μg/ml insulin, 1 % penicillin-streptomycin (Life Technologies) and

100ng/ml cholera toxin (Sigma-Aldrich). Stable cells were selected with 1 μg/μl puromycin (Sigma).

Transient transfections and infections

Transfections were carried out with Lipofectamine 2000 according to the manufacturer's protocol (Life Technologies) and adenoviral infections were carried out as previously described[2]. The Notch2 ICD adenoviral vector was kindly provided by Drs. Charles Eberhart and Xing Fan[3]. Control, HIF1α and HIF2α FlexiTube siRNA was purchased from QIAGEN (Hs_EPAS_5 and Hs_HIF1A_6).

DNA constructs and reagents

The NERT2 construct has been described previously[4] and was activated with 4-hydro-tamoxifen (Sigma) at a final concentration of 50nM. The HIF2α promoter luciferase reporter, containing an approximately 1.5 kb promoter element encompassing the region -1000 to +488 of the EPAS1/HIF2α transcription start site, was kindly provided by Drs. Tsaku Saito and Hiroshi Kawaguchi[5]. Cycloheximide was purchased from Cell signaling and actinomycin D from Sigma Aldrich. To block Notch signaling, cells were treated with 5-difluorophenylacetyl-L-alanyl-2-phenylglycine-1,1-di-methylethyl ester (DAPT; Calbiochem) at a final concentration of 2.5 μM, or DMSO as a control. The HIF1α inhibitor KC7F2 was used for 24 hours at a concentration of 40 μM.

Antibodies

The following primary antibodies were used in the study: anti-HIF2α (ab199, Abcam), anti-HIF2α (NB100-122, Novus Biologicals), anti-HIF1α (610958, BD Biosciences) and anti-β-actin (A2228, Sigma-Aldrich). Anti-Notch1 (D1E11), anti-Notch2 (D67C8), and anti-CSL (D10A4) were purchased from Cell Signaling Technology. As shown in Supplementary Figure 3B, we

utilized the Odyssey Imaging Systems from LI-COR, together with anti-mouse and anti-rabbit secondary antibodies that emit light at distinct wave-lengths, for simultaneous multiplex detection of HIF1α and HIF2α. The secondary antibodies utilized were: donkey-anti rabbit IRDye 800Cw (LI-COR Biosciences, utilized for HIF2α) and donkey-anti mouse IRDye 680LT (LI-COR Biosciences, utilized for HIF1α and β-actin).

Chorioallantoic membrane assay

Fertilized chicken eggs were placed in an egg incubator (De Rycke Savimat MG200) under rotation at 37°C with 60% humidity on day one of embryonic development. On day three eggs were turned, taken off rotation, punctured with a small hole, and covered with adhesive tape. On day eight the holes were expanded and a small plastic ring (5-6 mm in diameter) was placed on top of the chorioallantoic membrane (CAM). 1-2 $x10^6$ DAOY cells of the indicated genotypes were suspended in 1:1 PBS and matrigel for a total volume of 30 µl/egg. The cell suspensions were transplanted inside the plastic ring on the CAM and the eggs were covered with parafilm. On day 13 the tumors were excised and fixed in 3% PFA for four hours at room temperature after which the tumors were dehydrated with ethanol series of 50%, 70%, 70% for one hour each. The tumors were visualized on a Zeiss SteREO Lumar V12 microscope with a 0.8x NeoLumar objective and weighed using an analytical laboratory scale.

Transcriptome analyses

Publicly available expression data sets were downloaded from Gene Expression Omnibus[6]. GSE16477, GSE29544 and GSE29544 were processed using the affy and limma package of R's (http://www.R-project.org) bioconductor library[7]. GSE36051 was processed as previously described[8]. Heatmaps were generated in R using the Heatplus package from the bioconductor library[7]. Correlation of mRNA expression levels in transcriptome data sets was carried out in R using the affy package and R's correlation test (using the Spearman method), or the GeneSapiens database, as described previously[8].

RNA sequencing of DAOY-NERT2, DAOY, $DAOY^{Notch1-/-}$ and $DAOY^{Notch2-/-}$ cells was performed using the TrueSeq RNA kit from Illumina according to the manufacturer's protocol. The quality of the cDNA libraries was tested on an Agilent 2100 bioanalyzer. The libraries from the DAOY-NERT2 cells were sequenced on an Illumina HiSeq 2000 system, and the reads were aligned to the human genome (assembly hg36) and a transcriptome database (RefSeq and Ensembl) using bowtie [9]. RPKM values were generated using rpkmforgenes[10]. Up-regulated genes were selected using a fold change of minimum two and a cut off value of 5 rpkm. For the DAOY-NERT2 transcriptomes, HIF2α genes were defined as genes that were up-regulated by TMX treatment and downregulated at least 1.5 fold with HIF2α siRNA, but not HIF1α siRNA treatment. To explore enriched GO terms, lists of differential expressed genes were uploaded to DAVID v6.7 and analyzed using the Functional annotation chart and a P-value/ESEA score less than 0.05[11].

For the $DAOY^{Notch1-/-}$ and $DAOY^{Notch2-/-}$ transcriptomes, libraries were constructed using Illumina TruSeq Stranded mRNA protocol (poly-A-selection). Clustering was carried out using 'cBot' and samples were sequenced on HiSeq2500 (HiSeq Control Software 2.2.58/RTA 1.18.64) with a 1x51 setup using 'HiSeq SBS Kit v4' chemistry. The Bcl to FastQ conversion was performed using bcl2fastq_2.17 from the CASAVA software suite. The quality scale used is Sanger / phred33 / Illumina 1.8+. All libraries contained a minimum number of 20.21M reads and were aligned to reference genome Homo sapiens, GRCh37. Data were processed by the standard NGI-RNAseq pipeline from SciLifeLab (https://github.com/SciLifeLab/NGI-RNAseq/blob/master/docs/output.md). Briefly, mapped reads were normalized to Fragments Per Kilobase of transcript per Million mapped reads (FPKM) with StringTie[12,13]. Fold analysis was performed to identify deregulated genes with FPKM fold change > 2. The transcriptome data in Figure 4 and 5 are deposited at Gene Expression Omnibus (GSE IDs: 113353 and 113753).

CRISPR/Cas9 genome editing

The generation of HIF2α, CSL, Notch1 and Notch2 gene knockout DAOY cell lines using *CRISPR/Cas9* was carried out in accordance with procedures previously described[14]. The gRNA sequences used were, HIF2α: 5´-GATTGCCAGTCGCATGATGG-3´; CSL: 5'-

AAACATTGTATATATCTGAC-3´; Notch1: 5´- GGTGAGACCTGCCTGAATGG-3’; Notch2: 5´-GGTGGAGCCTGGAGTACAGG-3´. DAOY cells were transfected with the guide RNA Cas9 vector and puromycin at 1µg/ml was used for selection. Single cell colonies were isolated and subjected to Western blot analysis. The HIF2α sequence targeted by the CRISPR construct was amplified by PCR, using 40 ng of genomic DNA and a KOD DNA Polymerase (Merck Millipore). The purified PCR product was sequenced. PCR primers were as follows: Forward: 5´-AGGAGACGGAGGTGTTCTATGA-3´; Reverse: 5´- ACTCATGATGGTGTTTCTGTGG-3´.

ChIP-seq analysis

a. CSL ChIP

MDA-MB-231 cells were cultivated for 48h on Fc or Jagged1-Fc coated plates and chromatin was isolated from 90% confluent plates ($6x10^6$ cells/IP) using a commercially available ChIP enzymatic kit (SimpleChIP Enzymatic Chromatin IP Kit, CST, Danvers, USA) according to the manufacturer's instructions with the following changes: Before immunoprecipitation (IP), the chromatin was diluted 1:10 with ChIP dilution buffer (Millipore, Merck) and pre-cleared using Protein A/G beads (Pierce ChIP-grade Protein A/G Magnetic Beads, ThermoFisher Scientific, Waltham, USA) by incubation for 3h at 4°C on an end-to-end rotator. Beads were removed and chromatin was incubated over night at 4°C on an end-to-end rotator with anti-RBPSUH antibody (Cell Signaling Technology). As a control, a cell line deficient for CSL (MDA-MB-231 CSL-KO)[14] was utilized. The next day, chromatin/protein complexes were incubated with Protein A/G beads (Pierce ChIP-grade Protein A/G Magnetic Beads, ThermoFisher Scientific) for 4h at 4°C on an end-to-end rotator. Beads were then washed at 4°C two times in low-salt washing buffer, four time in high-salt washing buffer, two times in LiCl washing buffer and two times in TE buffer (all washing buffers were bought from Millipore, Merck).

After chromatin elution, DNA was purified using ChIP DNA purification columns (ChIP DNA Clean & Concentrator, Zymogen, Irvine, USA). DNA quality was assessed using a Bioanalyzer (HS DNA ChIP, Agilent Technologies, Santa Clara, USA) and DNA concentration was determined using Qubit (ThermoFisher Scientific). Raw DNA samples were then submitted to library preparation at Science for Life laboratories (SciLife, Stockholm, Sweden).

b. ChIP-seq analysis

ChIP libraries were constructed using Rubicon ThruPLEX (Rubicon genomics, Ann Arbor, USA), with no fragmentation option. Clustering was done by 'onboard clustering' and samples were sequenced on HiSeq2500 (HiSeq Control Software 2.2.58/RTA 1.18.64) with a 1x51 setup using 'HiSeq Rapid SBS Kit v2' chemistry. The Bcl to FastQ conversion was performed using bcl2fastq from the CASAVA software suite. The quality scale used is Sanger / phred33 / Illumina 1.8+. All libraries contained a minimum number of 13.84M reads and were aligned to reference genome Homo sapiens, GRCh37.

Peak detection, motif analysis and genome browser file generation of ChIP libraries were performed using HOMER (Hypergeometric Optimization of Motif EnRichment) NGS software suite[15] with the respective input as baseline. Peaks were identified using the following parameters: Fold over input required = 4.00, Poisson p-value over input required = 1.00e-04, FDR rate threshold = 0.001, size of region used for local filtering = 10000, Poisson p-value over local region required = 1.00e-04, Fold over local region required = 4.00.

c. *RNA-seq library preparation*

Total RNA was isolated from 90 % confluent plates of MDAMB231 cells that had been cultivated on Fc or Jagged1-Fc coated plates for 48h. RNA was purified using the *RNeasy Mini* Kit (Qiagen, Hilden, Germany), according to the manufacturer's instructions. RNA concentration and quality was assessed by Qubit (ThermoFisher Scientific) and RNA integrity number (RIN) was calculated by using the *Agilent RNA 6000 Nano* Kit (Agilent Technologies). Two biological replicates for each RNA sample were subjected to cDNA library preparations at Science for Life laboratories (SciLife, Stockholm, Sweden).

RNA libraries were constructed using Illumina TruSeq Stranded mRNA protocol (poly-A-selection). Clustering was carried out using 'cBot' and samples were sequenced on HiSeq2500 (HiSeq Control Software 2.2.58/RTA 1.18.64) with a 1x51 setup using 'HiSeq SBS Kit v4' chemistry. The Bcl to FastQ conversion was performed using bcl2fastq_2.17 from the CASAVA software suite. The quality scale used is Sanger / phred33 / Illumina 1.8+. All

libraries contained a minimum number of 19.08M reads and were aligned to reference genome Homo sapiens, GRCh37.

HOMER (Hypergeometric Optimization of Motif EnRichment) NGS software suite[15] was used for the generation of genome browser files and for counting the number of raw reads in each sample. Differential gene expression analysis was performed with edgeR[16]. Differentially expressed genes with statistical significance were identified with $p \leq 0.05$, log2-fold change higher than 1.5 and a false discovery rate lower than 0.05.

References

1 Debnath J, Muthuswamy SK, Brugge JS. Morphogenesis and oncogenesis of MCF-10A mammary epithelial acini grown in three-dimensional basement membrane cultures. *Methods* 2003; **30**: 256–268.

2 Chapman G, Liu L, Sahlgren C, Dahlqvist C, Lendahl U. High levels of Notch signaling down-regulate Numb and Numblike. *J Cell Biol* 2006; **175**: 535–40.

3 Fan X, Mikolaenko I, Elhassan I, Ni X, Wang Y, Ball D *et al.* Notch1 and Notch2 Have Opposite Effects on Embryonal Brain Tumor Growth. *Cancer Res* 2004; **64**: 7787–7793.

4 Schroeder T, Just U. Notch signalling via RBP-J promotes myeloid differentiation. *EMBO J* 2000; **19**: 2558–2568.

5 Saito T, Fukai A, Mabuchi A, Ikeda T, Yano F, Ohba S *et al.* Transcriptional regulation of endochondral ossification by HIF-2alpha during skeletal growth and osteoarthritis development. *Nat Med* 2010; **16**: 678–686.

6 Edgar R, Domrachev M, Lash AE. Gene Expression Omnibus: NCBI gene expression and hybridization array data repository. *Nucleic Acids Res* 2002; **30**: 207–10.

7 Gentleman R, Carey V, Bates D, Bolstad B, Dettling M, Dudoit S *et al.* Bioconductor: open software development for computational biology and bioinformatics. *Genom* 2004; **5**: R80.

8 Jin S, Mutvei AP, Chivukula I V, Andersson ER, Ramsköld D, Sandberg R *et al.* Non-canonical Notch signaling activates IL-6/JAK/STAT signaling in breast tumor cells and is controlled by p53 and IKKα/IKKβ. *Oncogene* 2012; : 1–11.

9 Langmead B, Trapnell C, Pop M, Salzberg SL. Ultrafast and memory-efficient alignment of short DNA sequences to the human genome. *Genome Biol* 2009; **10**: R25.

10 Ramsköld D, Wang ET, Burge CB, Sandberg R. An abundance of ubiquitously expressed genes revealed by tissue transcriptome sequence data. *PLoS Comput Biol* 2009; **5**: 1–11.

11 Huang DW, Sherman BT, Lempicki R a. Systematic and integrative analysis of large gene lists using DAVID bioinformatics resources. *Nat Protoc* 2009; **4**: 44–57.

12 Dobin A, Davis CA, Schlesinger F, Drenkow J, Zaleski C, Jha S *et al.* STAR: Ultrafast universal RNA-seq aligner. *Bioinformatics* 2013; **29**: 15–21.

13 Pertea M, Pertea G, Antonescu C, Chang T, Mendell J, Salzberg S. StringTie enables improved reconstruction of a transcriptome from RNA-seq reads. *Nat Biotechnol* 2015; **33**: 290–295.

14 Braune E-B, Tsoi YL, Phoon YP, Landor S, Silva Cascales H, Ramsköld D *et al.* Loss of CSL Unlocks a Hypoxic Response and Enhanced Tumor Growth Potential in Breast Cancer Cells. *Stem Cell Reports* 2016; **6**. doi:10.1016/j.stemcr.2016.03.004.

15 Heinz S, Benner C, Spann N, Bertolino E, Lin YC, Laslo P *et al.* Article Simple Combinations of Lineage-Determining Transcription Factors Prime cis -Regulatory Elements Required for Macrophage and B Cell Identities. *Mol Cell* 2010; **38**: 576–589.

16 Robinson MD, Mccarthy DJ, Smyth GK. edgeR : a Bioconductor package for differential expression analysis of digital gene expression data. *Bioinformatics* 2010; **26**: 139–140.
