# Supplementary figures and images for: Notch signaling promotes a HIF2α-driven hypoxic response in multiple tumor cell types
Source: Oncogene. 2018 Jul 11;37(46):6083–95. doi: 10.1038/s41388-018-0400-3 (PMC6237764; doi:10.1038/s41388-018-0400-3)

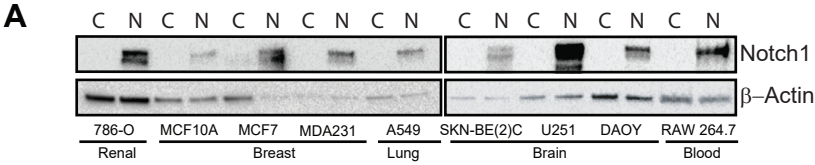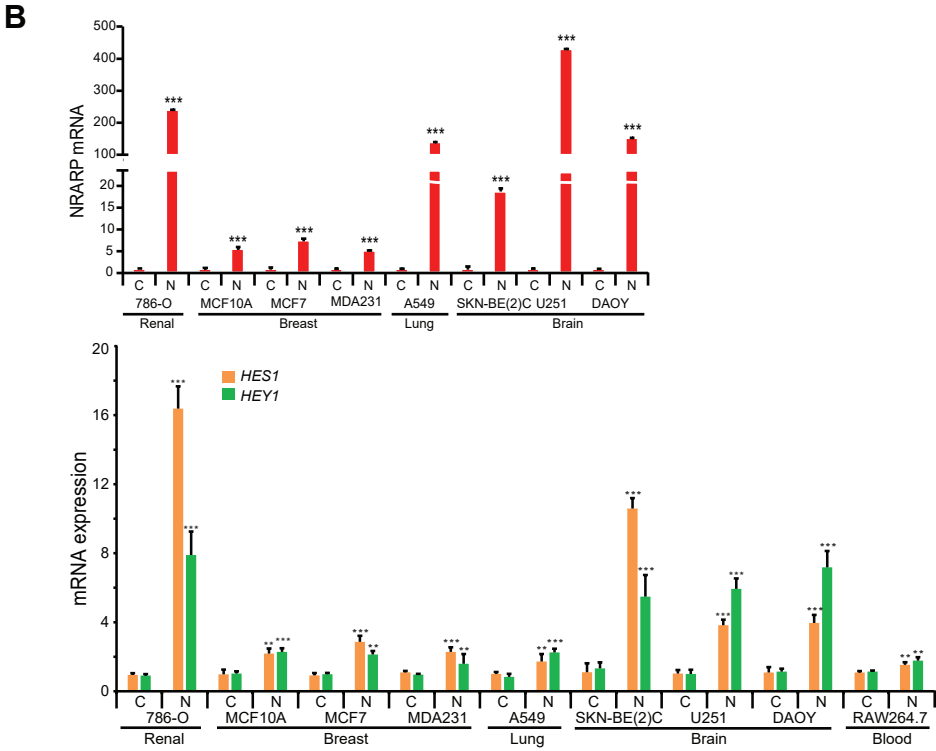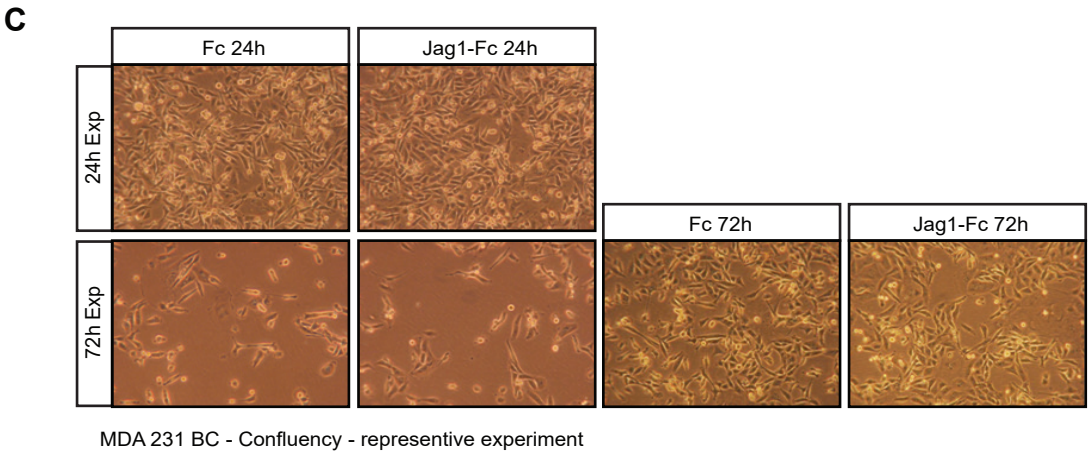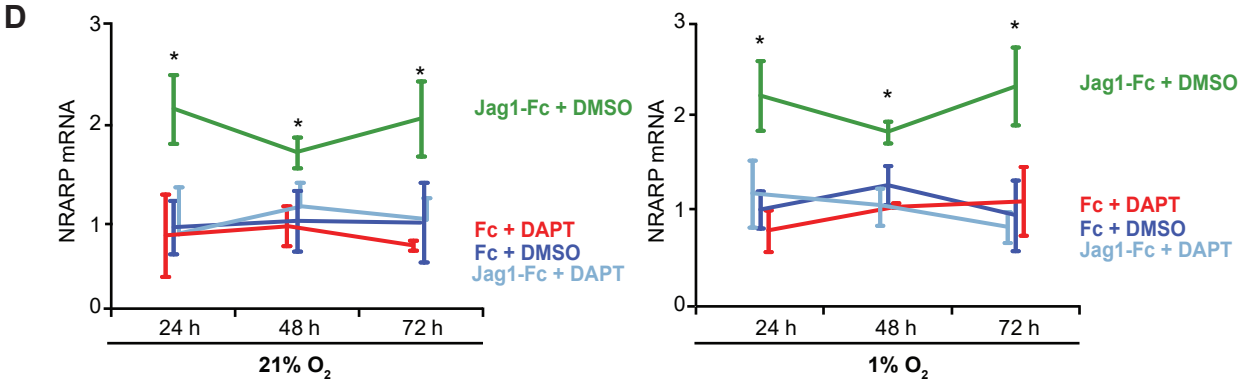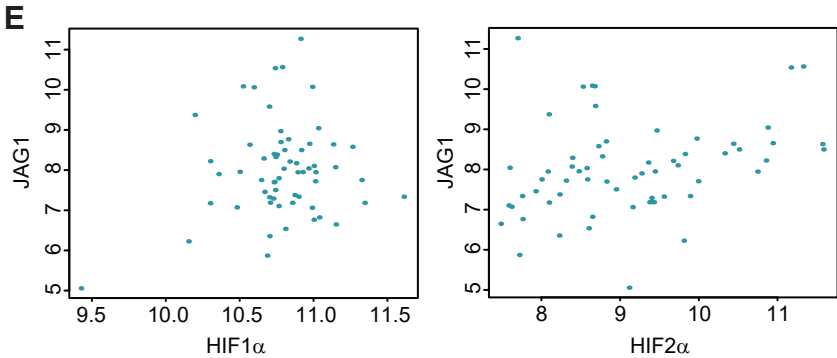

| Correlation JAG1 VS:  | HIF1α |      | HIF2α |        |
|-----------------------|-------|------|-------|--------|
|                       | r     | p    | r     | p      |
| Medulloblastoma, n=62 | 0.04  | 0.73 | 0.36  | 0.0043 |

Sup. Figure 1

Supplement: Supplementary file 2 — Supplementary Figure 1 [file 41388_2018_400_MOESM2_ESM.pdf]

**A**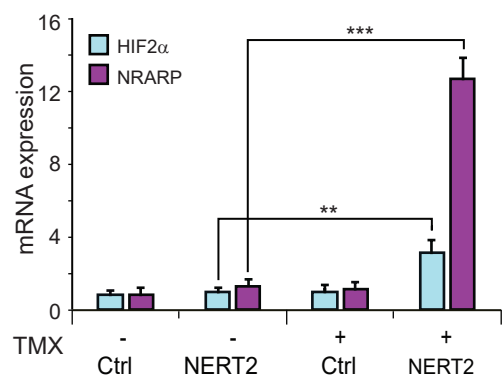**B**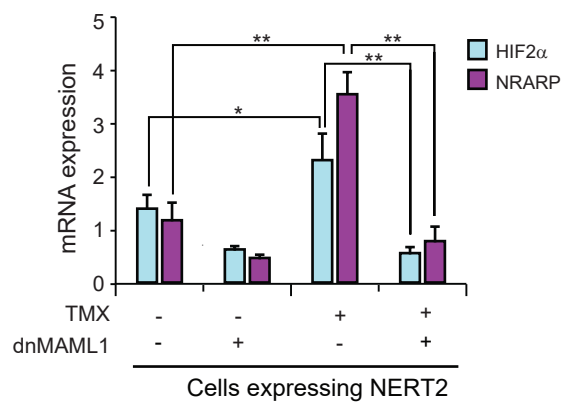**C**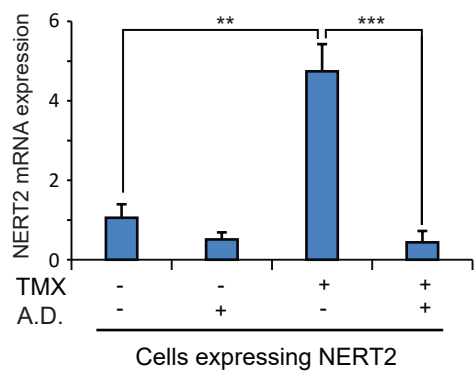**D**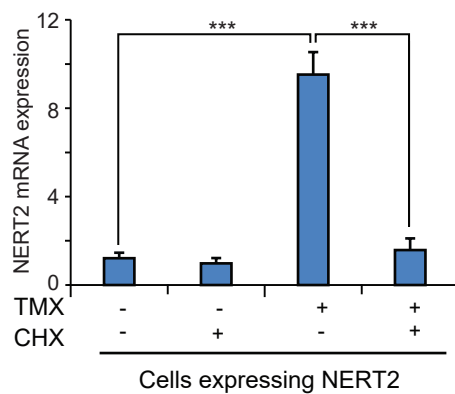**E**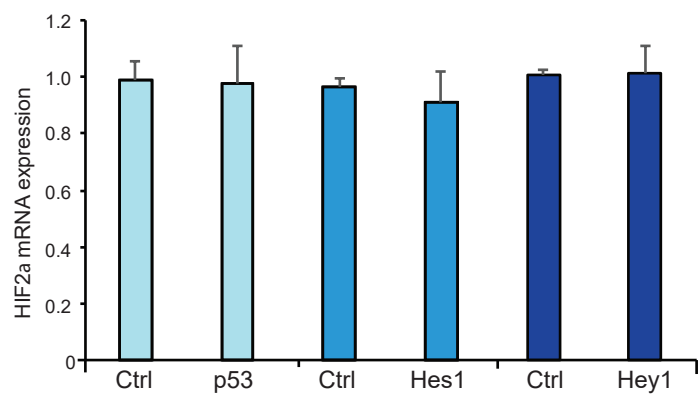

Sup. Figure 2

Supplement: Supplementary file 3 — Supplementary Figure 2 [file 41388_2018_400_MOESM3_ESM.pdf]

**A**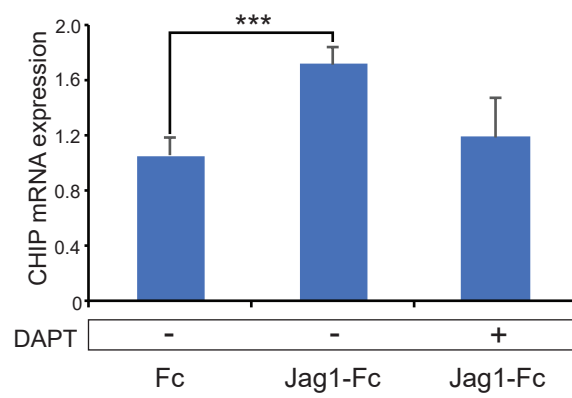**B**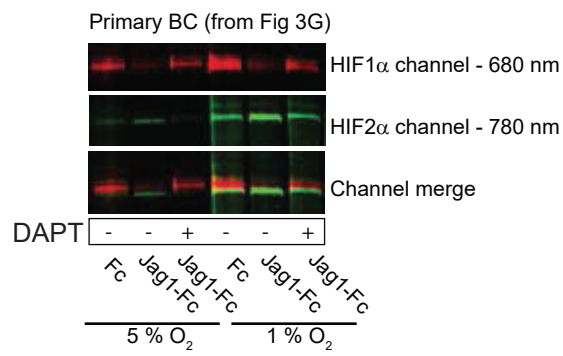

Supplement: Supplementary file 4 — Supplementary Figure 3 [file 41388_2018_400_MOESM4_ESM.pdf]

**A**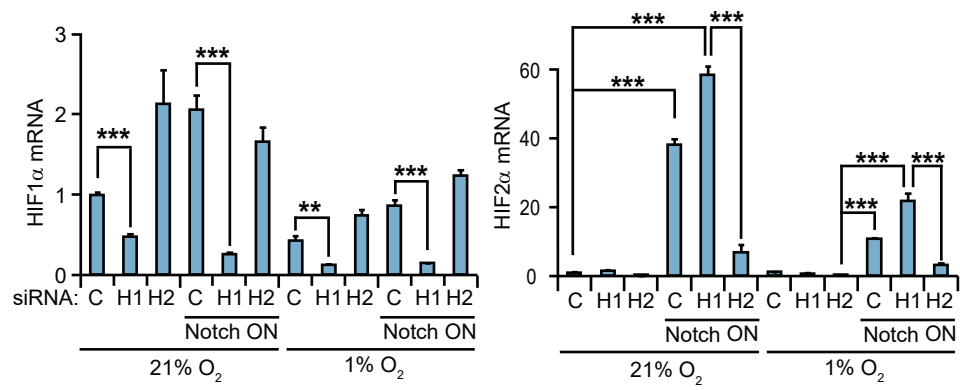**B**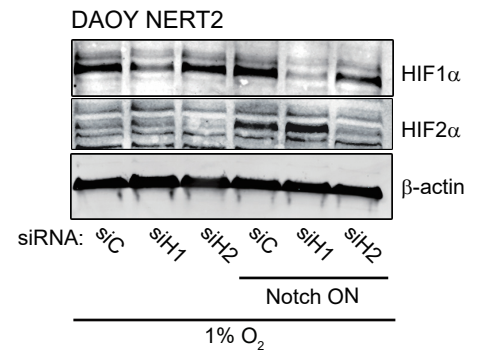**C**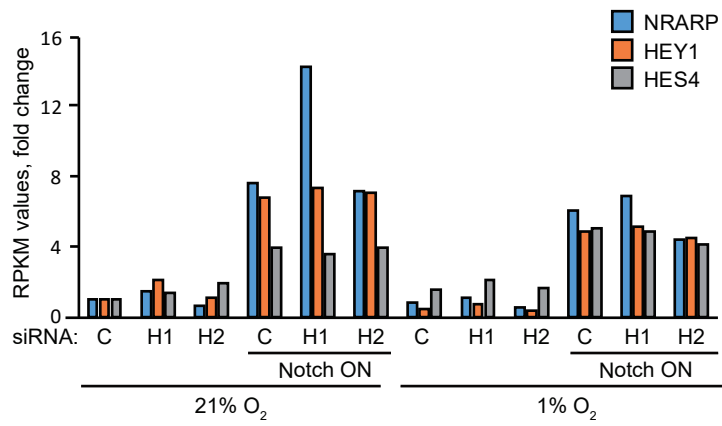

Sup.Figure 4

Supplement: Supplementary file 5 — Supplementary Figure 4 [file 41388_2018_400_MOESM5_ESM.pdf]
