# Supplementary material for: Notch signaling promotes a HIF2α-driven hypoxic response in multiple tumor cell types
Source: Oncogene. 2018 Jul 11;37(46):6083–95. doi: 10.1038/s41388-018-0400-3 (PMC6237764; doi:10.1038/s41388-018-0400-3)
Supplement: Supplementary file 6 — Supplementary Figure 5 [file 41388_2018_400_MOESM6_ESM.pdf]

	Human primers	
HIF1alpha	TTCCAGTTACGTTCCTTCGATCA	TTTGAGGACTTGCGCTTTCA
HIF2alpha	TGCTCCCACGGCCTGTAC	TTGTCACACCTATGGCATATCACA
ID3	GAGCTTGCTGGACGACATGAA	ATGACGCGCTGTAGGATTTCC
TGFB2	GTCAGGCCATATCCATCAAGGA	GCAGTCAAGCTGGATGGAAACT
NOTCH3	AGTTACCCCCAAGAGGCAAGT	TATCTCGGTCACGCTGCAA
CPE	CAGTACCCGCATTCACATCATG	TGCTTCGACCCACAAACCA
b-actin	CCAGAGGCGTACAGGGATAG	CCAACCGCGAGAAGATGA
NRARP	TTCTCCCTCTCCCTCAAATCC	AACTGCAAAACAAGCCGGTT
18S	AGAGGTGAAATTCTTGGACCGG	ATCGTCTTCGAACCTCCGACTT
WISP1	TGCTGTAAGATGTGCGCTCAG	ACACTCCTATTGCGTACCTCG
HCLS1	AGTGGGCCATGATGTGTCTG	CTCCCCATCGTTGCTCCTTT
EEF1A2	GAAGACCCACATCAACATCGT	CTCCGCATTTGTAGATGAGGTG
ITGBL1	AGACCTACGACGGGAGCAC	ACCTGCATTAGAGCAGATGATGT
GDA	GCTGGAAGTAGCATAGACCTGC	TCTTCTGCAAAGTCGATGTTCTG
ITGB4	GCAGCTTCCAAATCACAGAGG	CCAGATCATCGGACATGGAGTT
DNER	AAGGCTATGAAGGTCCCAACT	CTGAGAGCGAGGCAGGATTT
PID1	TCCTGGAAATCCGGCCATTC	AGGTCATCATTGATCTCCCTGT
NERT	TGCCTGGCTAGAGATBCCTGATG	TTTCCCTGGTTCCTGTCCAAG
HES1	TCGTTCATGCACTCGCTGA	AGGCGGACATTCTGGAAATG
HEY1	AAAAAGCCGAGATCCTGCAGA	CCGAAATCCCAAACTCCGATA
CHIP	AGCAGGGCAATCGTCTGTTC	CAAGGCCCGGTTGGTGTAATA
HES4	CGCTCAGCTCAAAACCCTCAT	AGGTGTCTCACGGTCATCTCCA
	Mouse Primers	
HIF1alpha	GAATGCTCAGAGGAAGCGAAA	ACAGTCACCTGGTTGCTGCA
HIF2alpha	CCAACTCTTTGCTCCGGTTTT	GGCATTACCAGACAGCTTCGA
Hes1	AGAGGCTGCCAAGGTTTTTG	ACTGGAAGGTGACACTGCGTT
Hey1	CTTGAGTTCGGCGCTGTGTTCC	GATGCCTCTCCGTCTTTTCCT
GAPDH	AGGAGCGAGACCCCACTAACAT	GTGAAGACACCAGTAGACTCCACG

Sup. Figure 5
