# Supplementary material for: Notch signaling promotes a HIF2α-driven hypoxic response in multiple tumor cell types
Source: Oncogene. 2018 Jul 11;37(46):6083–95. doi: 10.1038/s41388-018-0400-3 (PMC6237764; doi:10.1038/s41388-018-0400-3)
Supplement: Supplementary file 7 — Supplementary Table 1 [file 41388_2018_400_MOESM7_ESM.pdf]

BP_FAT GO Term	Genes (n)	P-value	Benjamini
cell surface receptor linked signal transduction	35	1,50E-03	1,30E-01
regulation of cell proliferation	25	4,50E-06	3,40E-03
cell adhesion	17	4,20E-03	2,50E-01
biological adhesion	17	4,20E-03	2,40E-01
positive regulation of cell proliferation	15	1,80E-04	3,90E-02
behavior	14	1,90E-03	1,50E-01
response to organic substance	13	9,70E-02	8,30E-01
blood vessel development	12	7,90E-05	3,00E-02
vasculature development	12	9,80E-05	3,00E-02
positive regulation of signal transduction	12	4,00E-04	6,60E-02
positive regulation of cell communication	12	9,80E-04	1,10E-01
negative regulation of cell proliferation	12	2,10E-03	1,50E-01
regulation of phosphorylation	12	1,30E-02	4,70E-01
cell motion	12	1,50E-02	5,00E-01
regulation of phosphate metabolic process	12	1,80E-02	5,10E-01
regulation of phosphorus metabolic process	12	1,80E-02	5,10E-01
vesicle-mediated transport	12	5,10E-02	7,20E-01
regulation of cell adhesion	11	2,50E-06	3,90E-03
blood vessel morphogenesis	11	1,10E-04	2,70E-02
cell morphogenesis	10	1,60E-02	5,00E-01
cellular component morphogenesis	10	3,00E-02	6,10E-01
neuron differentiation	10	5,10E-02	7,20E-01
locomotory behavior	9	1,10E-02	4,20E-01
skeletal system development	9	2,40E-02	5,70E-01
chordate embryonic development	9	2,90E-02	6,20E-01
embryonic development ending in birth or egg hatching	9	3,00E-02	6,10E-01
cell projection organization	9	4,90E-02	7,20E-01
Wnt receptor signaling pathway	8	6,40E-04	8,60E-02
angiogenesis	8	1,20E-03	1,20E-01
taxis	8	1,90E-03	1,60E-01
chemotaxis	8	1,90E-03	1,60E-01
regulation of cell migration	8	2,60E-03	1,70E-01
regulation of locomotion	8	5,20E-03	2,70E-01
regulation of cell motion	8	5,30E-03	2,70E-01
cell migration	8	3,20E-02	6,10E-01
cell motility	8	5,20E-02	7,20E-01
localization of cell	8	5,20E-02	7,20E-01
negative regulation of molecular function	8	7,40E-02	8,00E-01
enzyme linked receptor protein signaling pathway	8	8,20E-02	8,20E-01

Sup. Table 1
